# Ultra High-Speed Radio Frequency Switch Based on Photonics

**DOI:** 10.1038/srep17263

**Published:** 2015-11-26

**Authors:** Jia Ge, Mable P. Fok

**Affiliations:** 1 Lightwave and Microwave Photonic Laboratory, College of Engineering, The University of Georgia.

## Abstract

Microwave switches, or Radio Frequency (RF) switches have been intensively used in microwave systems for signal routing. Compared with the fast development of microwave and wireless systems, RF switches have been underdeveloped particularly in terms of switching speed and operating bandwidth. In this paper, we propose a photonics based RF switch that is capable of switching at tens of picoseconds speed, which is hundreds of times faster than any existing RF switch technologies. The high-speed switching property is achieved with the use of a rapidly tunable microwave photonic filter with tens of gigahertz frequency tuning speed, where the tuning mechanism is based on the ultra-fast electro-optics Pockels effect. The RF switch has a wide operation bandwidth of 12 GHz and can go up to 40 GHz, depending on the bandwidth of the modulator used in the scheme. The proposed RF switch can either work as an ON/OFF switch or a two-channel switch, tens of picoseconds switching speed is experimentally observed for both type of switches.

A radio frequency (RF) switch, also called a microwave switch, is a device to route high frequency signals between different transmission channels or devices, which is an essential component in a wide range of applications including wireless communications, radar systems, satellite communications, and microwave test systems. Switching speed is a key parameter to define a RF switch, which is the time needed to change the state of a switch from ON to OFF or from OFF to ON. In conventional RF switches, the switching speed is limited to microsecond range for both electromechanical switches and micro-electromechanical systems (MEMS) switches and nanosecond range for solid-state switches^1^,^2^,^3^,^4^. The speed is either limited by the physical properties of the materials and switching mechanisms of the electrical devices or the limited tuning capability of the resistor inductor capacitor (RLC) circuits that is being utilized in the switch.

Photonics technology is inherently high-speed, broadband, low loss, and electro-magnetic immune, and it has been proved to be a promising way to break the bottlenecks of various fields, especially in the area of microwave^5^,^6^,^7^,^8^,^9^. By taking advantages of the unique properties from photonics, the performances and capabilities of microwave components and microwave systems are significantly enhanced. Existing microwave photonics (MWP) developments including RF filters^10^,^11^,^12^,^13^,^14^,^15^,^16^,^17^,^18^,^19^,^20^,^21^,^22^,^23^,^24^,^25^,^26^,^27^, phase shifter^28^, delay line^29^, arbitrary waveform generation^30^, and frequency measurement^31^. MWP offers unique characteristics to the above systems, such as high-speed operation, flexible tunability and reconfigurability, and wide operation bandwidth. Although tuning of MWP system at tens of picosecond speed has been demonstrated for various systems^29^,^34^, implementing RF switches with photonics has not been studied and switching RF signals at tens of picosecond speed has never been demonstrated.

In this paper, we draw inspiration from our previous work on high-speed tunable microwave photonic filter^9^,^10^ and propose an ultra high-speed RF switch based on photonics technology, achieving an RF switch with switching speed in the range of tens of picosecond, i.e. hundreds times faster than existing technologies. The proposed MWP RF switch can work as an ON/OFF switch that turns the input RF signal ON and OFF, as well as a two-channel switch that switches one of the two RF signals with different frequencies to the output according to the control signal. The high-speed switching capability of the MWP RF switch is achieved by the mean of a high-speed tunable MWP notch filter, which is rapidly tuned to block the unwanted signal while allowing the desired one to pass through, essentially switching out only the desired signal. Switching speed of the MWP RF switch is governed by the tuning speed of the MWP filter. Previously, we have demonstrated a MWP notch filter with tens of gigahertz tuning speed^10^,^11^, which is the fastest among approaches that have been reported during the last decade^13^, including the use of mirroring heaters^17^,^18^,^19^, tunable delay lines^23^,^24^,^25^, mechanical tuning^22^,^26^ or taps wavelength tuning^12^,^20^,^27^. More recently, an optical comb based MWP filter with tuning speed of 40 ns has been demonstrated with the use of an electrical tunable phase shifter^12^, while the use of a photonics crystal delay line based MWP filter can potentially achieve tuning speed of in nanosecond range^13^. Thus, with the development of our MWP notch filter with tens of picosecond tuning speed, an ultra high-speed MWP RF switch is achieved based on the fast electro-optics Pockels effect utilized in the filter. Moreover, the proposed MWP RF switch has a high isolation over 50 dB, and supports signals with carrier frequencies from DC to tens of gigahertz governed by the modulation bandwidth during electrical-to-optical conversion.

## High-speed Continuously Tunable MWP Notch Filter

**Figure 1:**
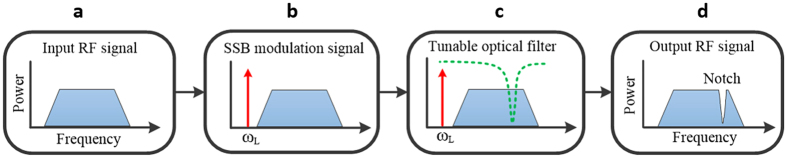
Operating principle of the tunable MWP notch filter.

Figure 1 illustrates the principle of our previously demonstrated MWP notch filter we used for the MWP RF switch. Assume an input RF signal has a frequency spectrum as shown in Fig. 1a, it is first up-converted to the optical frequency range by modulating the RF signal onto an optical carrier through single-sideband (SSB) modulation in an electro-optic intensity modulator, the corresponding optical frequency spectrum is shown in Fig. 1b. There is a direct correspondence between the RF spectrum and the optical frequency spectrum, where the red arrow indicates the optical carrier (f_c_) that is at around 193.4 THz (corresponds to 1550 nm in wavelength), while the blue area is the SSB spectrum that represents the input RF signal. A tunable optical notch filter we developed^32^ is used to manipulate the optical spectrum such that the unwanted spectral components are removed from the optical spectrum, as shown in Fig. 1c. The modified optical spectrum is then down-converted back to an RF signal by beating between the optical carrier and the SSB spectrum at a photodetector. The resultant frequency spectrum of the filtered RF signal is shown in Fig. 1d, which part of the spectrum has been filtered out by the MWP notch filter. Thus, the notch frequency of the MWP notch filter is tuned rapidly by adjusting the filter wavelength of the optical notch filter.

**Figure 2:**
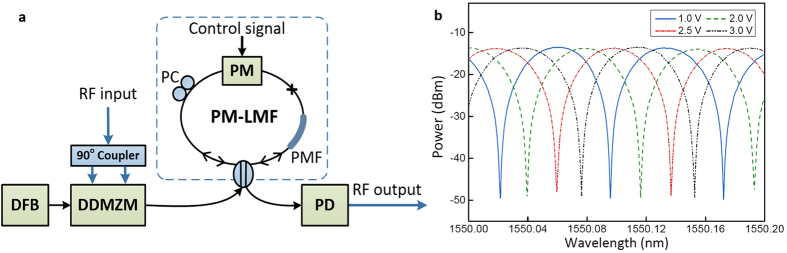
Demonstration of the high-speed tunable MWP notch filter. (**a**) Experimental setup of the PM-LMF based MWP notch filter. DFB: distributed feedback laser; DDMZM: dual-drive Mach-Zehnder modulator; PM: phase modulator; PMF: polarization maintaining fiber; PC: polarization controller; PD: photo detector. (**b**) Measured transmission optical spectra of the tunable PM-LMF at different tuning voltages.

The experimental setup of the MWP notch filter is shown in Fig. 2a. An input RF signal is modulated onto an optical carrier using a 12-GHz dual-drive Mach-Zehnder modulator (DDMZM) via a 90-degree electrical hybrid coupler. A SSB modulated signal is generated by properly adjusting the biases of the upper and lower branches of the DDMZM. The generated SSB signal is then amplified and launched into a phase modulator based loop mirror filter (PM-LMF), as shown in the dashed box in Fig. 2a, through a 3-dB optical coupler for optical spectral filtering. The PM-LMF is used as a high-speed tunable optical notch filter for blocking the corresponding unwanted frequency portion of the RF signal from the SSB. The filtered optical signal is then detected by a photodetector and is converted back to an electrical RF signal. The resultant RF spectrum can be measured by an electrical spectrum analyzer, while the filter profile of the MWP notch filter is measured by an electrical network analyzer. Detailed specifications of major equipment and components used in the experiment can be found in the supplementary information.

The proposed PM-LMF has proven its fast and wide tunability in various applications^11^,^32^, its gigahertz tuning speed surpass all other existing optical filter techniques. As illustrated in the dashed box in Fig. 2a, the PM-LMF consists of a coupler, a piece of polarization maintaining fiber (PMF), a phase modulator (PM), and a polarization controller (PC). Its transmission function is described by the following equation,


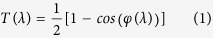


where φ(*λ*) depicts the phase difference between the two counter-propagating beams in the PM-LMF. The total phase difference is the accumulated phase difference induced by both the PM and PMF governed by the alignment between the PM and PMF. Equation (2) indicates the total phase difference when the transverse-electric (TE) axis of the PM is aligned with the fast axis of the PMF,


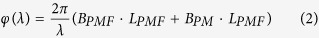


where *B*_*PMF*_, *L*_*PMF*_, *B*_*PM*_ and *L*_*PM*_ are the birefringence and length of the polarization maintaining fiber and phase modulator, respectively. The aligned PM and PMF work as a tunable birefringence device. The transmission function is determined by the birefringence and length of the PMF and the PM. During the tuning, all the parameters are kept constant except *B*_*PM*_, which is tuned based on electro-optics Pockels effect by applying different DC or AC voltages to the PM. Since Pockels effect has a fast response time, tens of GHz tuning speed is achieved in the PM-LMF and is mainly governed by the modulation bandwidth of the phase modulator.


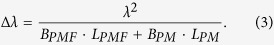


The free spectral range (FSR) of the PM-LMF is determined by equation (3), which also represents FSR of the tunable MWP notch filter. In the experiment, a 10-GHz phase modulator is used as the tuning device, which consists of a 71-mm LiNbO_3_ waveguide with a birefringence of 7.4 × 10^−3^ and 1-m of PMF pigtails with a birefringence of 3.0 × 10^−4^. By combining these with a piece of 37.5-m PMF with birefringence of 6.6 × 10^−4^, a MWP notch filter with FSR of 10 GHz is achieved. The FSR can be adjusted to meet specific application requirement by changing the length of the PMF, i.e. a longer piece of PMF results in a MWP filter with smaller FSR.

Measured transmission optical spectra of the PM-LMF (optical notch filter) at different tuning voltages are shown in Fig. 2b, measured by an optical spectrum analyzer with a resolution of 0.8 pm. A tunable optical comb filter with a FSR of 80 pm is observed, corresponding to a MWP notch filter with a FSR of 10 GHz. The peak-to-notch extinction ratios are over 35 dB, with both the spectral shape and extinction ratio remain unchanged during the entire tuning process. The PM-LMF is continuously tunable by applying different DC voltages to the PM, wavelength tuning over one FSR is observed at an applied voltage of 3.5 V and up to three FSR of tuning is recorded at an applied voltage of 5.5 V^32^. The change in comb spacing as a consequence of frequency tuning effect is less than 2 pm, thus its influence to both the MWP notch filter and the MWP RF switch is negligible. The PM-LMF is then used as an optical notch filter for achieving a MWP RF notch filter.

**Figure 3:**
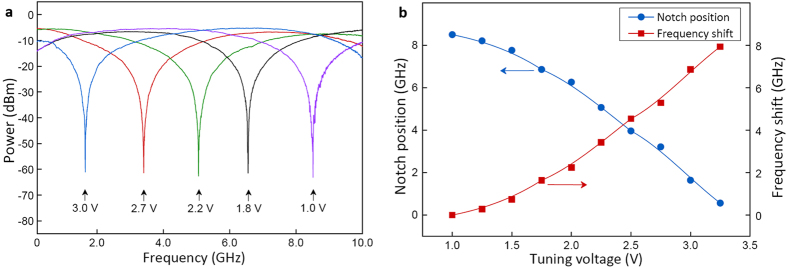
Rapid frequency tunability of the MWP notch filter. (**a**) Measured frequency tuning spectra of the MWP notch filter. (**b**) Notch position and frequency shift in response to different tuning voltages.

Figure 3a shows the frequency response of the MWP notch filter that is tuned over 0 to 10 GHz range. The frequency response is measured by an electrical network analyzer with an intermediate frequency bandwidth of 5 kHz. The 10-dB bandwidth of the MWP notch filter is 1.7 GHz, with an insertion loss of 6 dB at the transmission peak, while the notch has a 30-dB notch depth of 260 MHz wide. The notch rejection ratios are over 50 dB, providing good filter selectivity and isolation of the MWP switch. Stable filter profiles and constant notch rejection ratios are observed throughout the entire tuning range. Although the rejection ratio is susceptible to the accurate alignment between the optical notch and the RF frequency that is needed to be blocked, a wavelength tracking and control system can be used for optimization. The notch frequency of the MWP filter (blue circle curve) and the amount of notch frequency shift (red square curve) in response to different tuning voltages are shown in Fig. 3b. The notch frequency tuning is not obvious when applied voltage is under 1.0 V due to the insignificant change of birefringence in the phase modulator. The maximum tuning range is determined by the possible amount of birefringence change in the PM. In principle, the RF filter notch position can potentially be tuned over 30 GHz, according to the spectral tuning results of the PM-LMF in Fig. 2b. In Fig. 3a, a tuning range of 10 GHz is shown due to the bandwidth of the DDMZM we used in the experiment. It is worth noticing that since the half-wave voltage of the modulator is different for different driving frequencies at the phase modulator, the exact voltage needed for controlling the MWP switch depends on the required switching speed.

## Microwave Photonic Switch

**Figure 4:**
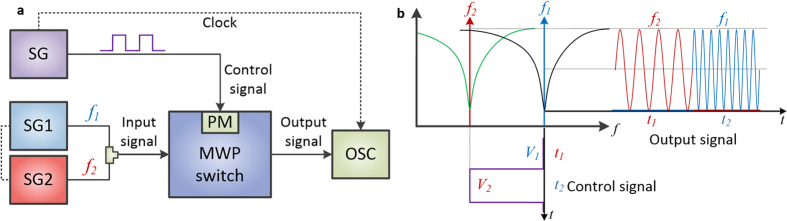
Schematic illustration of the high-speed MWP RF switch. (**a**) Experimental test setup of the MWP RF switch. SG: signal generator; PM: phase modulator; OSC: oscilloscope. (**b**) Operating principle of the MWP RF switch.

The proposed MWP RF switch is achieved using the fast tunable MWP notch filter developed above to rapidly switch out different inputs by blocking an unwanted signal and passing the desired signal, based on the ultrafast electro-optics Pockels effect. The experimental setup for testing the MWP RF switch is shown in Fig. 4a, and corresponding operating principle is illustrated in Fig. 4b. An input RF signal with frequencies at f_1_ and f_2_ is launched to the RF input of the MWP RF switch, while a square wave with two voltage levels (V_1_ and V_2_) is used as the control signal for switching out one of the input RF signal at a time. The voltage levels (V_control_) are set such that the MWP notch filter is aligned with frequency f_1_ when V_control_ = V_1_, resulting in the blocking of signal f_1_ and allowing f_2_ to pass through; while the MWP notch filter is aligned with f_2_ when V_control_ = V_2_, resulting in the blocking of signal f_2_ and allowing f_1_ to pass through. When the control signal is switching between V_1_ and V_2_, the output is also switching between f_2_ and f_1_.

The MWP RF switch can also serve as an ON/OFF switch when only one input RF signal is provided, such that the input RF signal is blocked when the notch position is located at the signal frequency, while it passes through the switch when the MWP notch filter is tuned away from the signal frequency by the control signal. Since the switching mechanism is based on birefringence change through electro-optic Pockels effect in the phase modulator, switching speed of the proposed RF switch is governed by the modulation bandwidth of the phase modulator as well as the switching speed of the control signal. To date, 40-GHz electro-optics phase modulators are commercially available with a response time of less than 25 ps.

**Figure 5:**
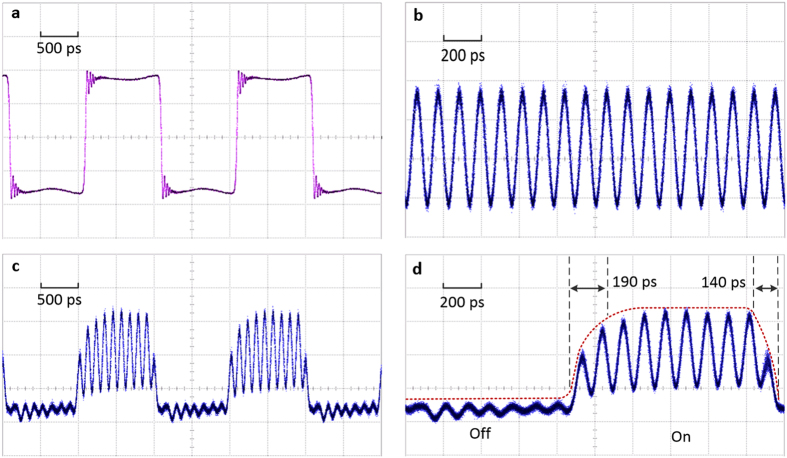
Measured ON/OFF switching performance of the proposed MWP switch. (**a**) 0.5 GHz square tuning signal. (**b**) 9 GHz sinusoidal input signal without tuning. (**c**) 9 GHz input signal is switched by the 0.5 GHz square tuning signal. (**d**) Close-up of the ON/OFF output signal (Vertical scales: 20 mV/div).

Figure 5 shows the ON/OFF switching performance of the proposed MWP RF switch, measured by a 30-GHz oscilloscope. A 0.5-GHz square wave is used as the control signal and a 9-GHz sinusoidal signal is launched into the DDMZM as the input RF signal, as shown in Fig. 5a,b, respectively. The MWP notch filter is first aligned at 9 GHz when no control signal is applied, i.e. the 9-GHz sinusoidal signal is blocked by the notch filter. The peak voltage of the control signal is set to 2.5 V such that the filter notch is tuned away from 9 GHz during the high voltage period of the square wave, allowing the input RF signal to completely pass through the RF switch. As a result of applying a 0.5-GHz square control signal to the RF switch, the input 9-GHz RF signal is switched ON and OFF periodically with an ON-state and OFF-state duration of 1000 ps, as shown in Fig. 5c. A closer look of the switching performance is shown in Fig. 5d. As shown, the input 9-GHz signal is completely blocked during the OFF state and is recovered and well maintained during the ON state. The switch has an ON-OFF transition time of ~140 ps and an OFF-ON transition time of ~190 ps, as shown by the dotted lines in Fig. 5d. Since the switching time is measured by taking the temporal separation of the closest signal peak of a fully recovered sinusoidal signal and an OFF state of the switch, thus, the real response time should be shorter than the measured value due to the “sampling” effect of the sinusoidal RF signal. Furthermore, the control signal itself has a rise and fall time of ~100 ps, which proposes a limitation in measuring the actual switching speed of the RF switch. Therefore, a shorter switching time could be obtained if a control signal with shorter rise/fall time is used. The power fluctuation during the ON/OFF transition period is caused by the significant high frequency ripples around the rising and falling edges of the control signal, while the weak residual input signal found during off state is due to misalignment between the filter notch and the input signal resulted from the non-flat low-level region in the control signal, as shown in Fig. 5a. Thus, a better square control signal with stable rising/falling edges and flat low-level region can be used to get better switching performance and suppression of the unwanted frequency.

**Figure 6:**
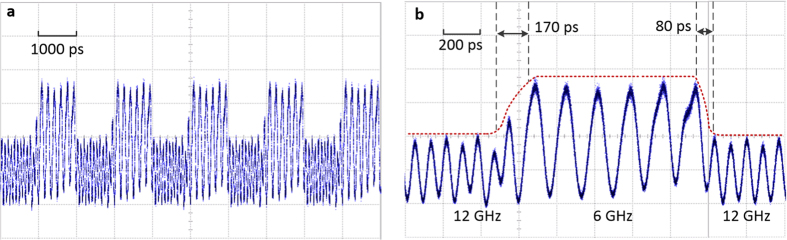
Measured two-channel switching performance of the proposed MWP switch. (**a**) Switching between 6 GHz and 12 GHz signals, tuned by a 0.5 GHz square tuning signal. (**b**) Close-up of the two-channel switching output signal (Vertical scales: 20 mV/div).

To demonstrate the capability to switch between two frequency channels, a set of 6-GHz and 12-GHz sinusoidal signals are used as the two input signals. Power of the 12-GHz signal is intentionally set to be weaker to enable an easy identification of the switching process. The switching performance is shown in Fig. 6. The two frequency signals are combined with a power combiner and launched into the MWP RF switch through the input port. The same 0.5-GHz square wave as shown in Fig. 5(a) is used as the control signal. The voltage levels of the square wave are set to 0 V and 2.8 V such that the MWP notch filter is blocking the 6-GHz signal at low voltage level and allowing the 12-GHz signal to pass through; while it is blocking the 12-GHz signal at high level and allowing the 6-GHz signal to pass through. At the output of the MWP RF switch, a RF signal that is switching between the two input RF signals (6 GHz and 12 GHz) is obtained as shown in Fig. 6a. As shown in the closer look in Fig. 6b, the output signal is a periodical signal switching between the 6-GHz and 12-GHz input RF signals. Similar to the ON/OFF switch performance, the switching times between different channels are ~170 ps (switching from 12 GHz to 6 GHz) and ~80 ps (switching from 6 GHz to 12 GHz). The above frequencies are chosen due to the synchronization requirement of the equipment used for the measurement. In principle, any frequency within the modulation bandwidth of the DDMZM can be used as the input signals, resulting in broadband operation. The presented results prove that the proposed MWP RF switch has a gigahertz switching speed, which is governed by the bandwidth of the phase modulator. The tens of picoseconds switching speed demonstrated above is based on a 10-GHz phase modulator, and can be potentially increased with the use of a larger bandwidth modulator. Since phase modulator with gigahertz bandwidth is integrable^33^, and most of the other devices are already available in integrated photonics platforms^13^,^34^, the miniaturization of the system could potentially be achieved with integrated photonic technology.

**Figure 7:**
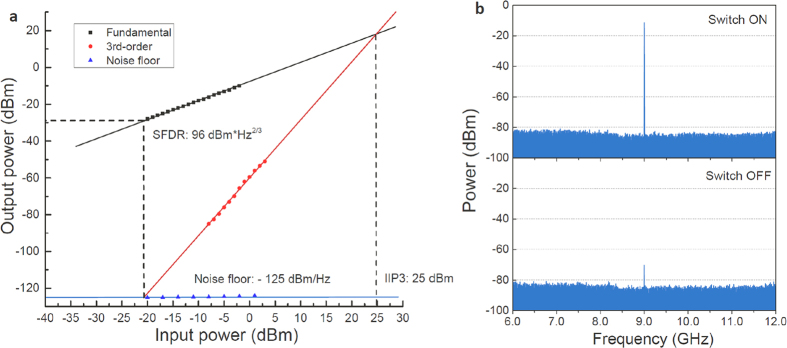
Performance metrics measurement of the MWP RF switch. (**a**) Dynamic range measurement of the system, with a SFDR of 96 dBm*Hz^2/3^ and IIP3 at 25 dBm. (**b**) Measured isolation between ON and OFF states, with an isolation of 58 dB.

Figure 7a shows the dynamic range measurement of the MWP RF switch. The system has a spurious free dynamic range (SFDR) of 96 dBm*Hz^2/3^, and the input third-order intercept point (IIP3) is at 25 dBm. A good linear response is observed between the input and output power, with a 1-dB compression point at 5 dBm, which is mainly governed by the RF amplifier used before the DDMZM. Insertion loss of the system is 6 dB during ON state. Figure 7b shows the measured frequency spectra at the switch output. The 9-GHz input signal found at the switch output is -12 dBm during ON state and is -70 dBm during OFF state, resulting in a switch isolation of 58 dB.

## Conclusion

In summary, a photonics based RF switch with fast switching speed and ultra-short ON/OFF transition time is proposed and experimentally demonstrated. The high-speed MWP RF switch makes use of a fast tunable MWP notch filter to block the unwanted channel and allow the desired channel to pass through, which is achieved by the ultra-fast electro-optics Pockels effect in a phase modulator. The MWP notch filter used in the RF switch can be continuously and rapidly tuned over a wide frequency range of tens of GHz, with a high rejection ratio of 50 dB. Both the ON/OFF switch and the two-channel switch have been experimentally demonstrated and shown a switching time of tens of picoseconds. This design significantly improves the switching speed to tens of picoseconds range as well as providing a stable and repeatable switching performances.

## Additional Information

**How to cite this article**: Ge, J. and Fok, M. P. Ultra High-Speed Radio Frequency Switch Based on Photonics. *Sci. Rep.* **5**, 17263; doi: 10.1038/srep17263 (2015).

## Supplementary Material

Supplementary Information

## References

[b1] Hindle P. The state of RF/Microwave switches. Microwave J. 53, 20–36 (2010).

[b2] Rebeiz G. M. RF MEMS: theory, design, and technology. John Wiley & Sons (2004).

[b3] Understanding RF/Microwave solid state switches and their applications, *Application Note, Agilent Technologies Agilent* (May, 2010). Available at: http://cp.literature.agilent.com/litweb/pdf/5989-7618EN.pdf.

[b4] Bacon P., Fischer D. & Lourens R. Overview of RF switch technology and applications. Microwave J. 57, 76–88 (2014).

[b5] Capmany J. & Novak D. Microwave photonics combines two worlds. Nature Photon. 1, 319–330 (2007).

[b6] Yao J. P. Microwave photonics. J. Lightwave Technol. 27, 314–335 (2009).

[b7] Capmany J. *et al.* Microwave photonic signal processing. J. Lightwave Technol. 31, 571–586 (2013).

[b8] Minasian R. A. Photonic signal processing of microwave signals. IEEE Trans. Microw. Theory Techn. 54, 832–846 (2006).

[b9] Capmany J. Ortega B. & Pastor. D. A tutorial on microwave photonic filters. J. Lightwave Technol. 24, 201 (2006).

[b10] Ge J., Feng H., Scott G. & Fok M. P. High-speed tunable microwave photonic notch filter based on phase modulator incorporated Lyot filter. Opt. Lett. 40, 48–51 (2015). 25531605 10.1364/OL.40.000048

[b11] Ge J., Feng H., Scott G. & Fok M. P. High-speed tunable microwave photonic notch filter based on phase modulator incorporated loop mirror filter. In *Optical Fiber Communication Conference, Optical Society of America*, W2A-61, Los Angeles, CA, doi:10.1364/OFC.2015.W2A.61 (March, 22, 2015).

[b12] Supradeepa V. R. *et al.* Comb-based radiofrequency photonic filters with rapid tunability and high selectivity. Nature Photon. 6, 186–194 (2012).

[b13] Sancho J. *et al.* Integrable microwave filter based on a photonic crystal delay line. Nat. Commun. 3, 1075 (2012). 23011129 10.1038/ncomms2092

[b14] Zhang W. & Minasian R. A. Switchable and tunable microwave photonic Brillouin-based filter. IEEE Photon. J. 4, 1443–1455 (2012).

[b15] Marpaung D., Morrison B., Pant R. & Eggleton B. J. Frequency agile microwave photonic notch filter with anomalously high stopband rejection. Opt. Lett. 38, 4300–4303 (2013). 24177078 10.1364/OL.38.004300

[b16] Loayssa A., Capmany J., Sagues M. & Mora J. Demonstration of incoherent microwave photonic filters with all-optical complex coefficients. IEEE Photon. Technol. Lett. 18, 1744–1746 (2006).

[b17] Marpaung D. *et al.* Si_3_N_4_ ring resonator-based microwave photonic notch filter with an ultrahigh peak rejection. Opt. Express 21, 23286–23294 (2013). 24104242 10.1364/OE.21.023286

[b18] Rasras M. S. *et al.* Demonstration of a tunable microwave-photonic notch filter using low-loss silicon ring resonators. J. Lightwave Technol. 27, 2105–2110 (2009).

[b19] Zhang D. *et al.* Tunable and reconfigurable bandstop microwave photonic filter based on integrated microrings and Mach–Zehnder interferometer. J. Lightwave Technol. 31, 3668–3675 (2013).

[b20] Dong J. *et al.* Compact notch microwave photonic filters using on-chip integrated microring resonators. IEEE Photon. J. 5, 5500307–5500307 (2013).

[b21] Chan E. H. W. & Minasian R. A. Widely tunable, high-FSR, coherence-free microwave photonic notch filter. J. Lightwave Technol. 26, 922–927 (2008).

[b22] Pastor D., Capmany J. & Ortega B. Broad-band tunable microwave transversal notch filter based on tunable uniform fiber Bragg gratings as slicing filters. IEEE Photon. Technol. Lett. 13, 726–728 (2001).

[b23] Kim T. Y., Oh C. K., Kim S. J. & Park C. S. Tunable photonic microwave notch filter with negative coefficient based on polarization modulation. IEEE Photon. Technol. Lett. 19, 907–909 (2007).

[b24] Mora J. *et al.* Photonic microwave tunable single-bandpass filter based on a Mach-Zehnder interferometer. J. Lightwave Technol. 24, 2500 (2006).

[b25] Hamidi E., Leaird D. E. & Weiner A. M. Tunable programmable microwave photonic filters based on an optical frequency comb. IEEE Trans. Microw. Theory Techn. 58, 3269–3278 (2010).

[b26] Mora J., Chen L. R. & Capmany J. Single-bandpass microwave photonic filter with tuning and reconfiguration capabilities. J. Lightwave Technol. 26, 2663–2670 (2008).

[b27] Mora J. *et al.* Automatic tunable and reconfigurable fiberoptic microwave filters based on a broadband optical source sliced by uniform fiber Bragg gratings. Opt. Express 10, 1291–1298 (2002). 19451991 10.1364/oe.10.001291

[b28] Liu W., Li W. & Yao J. An ultra-wideband microwave photonic phase shifter with a full 360 phase tunable range. IEEE Photon. Technol. Lett. 25, 1107–1110 (2013).

[b29] Bonjour R. *et al.* Continuously tunable true-time delays with ultra-low settling time. Opt. Express 23, 6952–6964 (2015). 25836915 10.1364/OE.23.006952

[b30] Cundiff S. T. & Weiner A. M. Optical arbitrary waveform generation. Nature Photon. 4, 760–766 (2010).

[b31] Chi H., Zou X. & Yao J. An approach to the measurement of microwave frequency based on optical power monitoring. IEEE Photon. Technol. Lett. 20, 1249–1251 (2008).

[b32] Ge J., Feng H. & Fok M. P. High-speed wavelength tunable DPSK demodulation using a phase modulator based loop mirror filter. Opt. Lett. 39, 3500–3503 (2014). 24978521 10.1364/OL.39.003500

[b33] Lu H. *et al.* 6-micron interaction length electro-optic modulation based on lithium niobate photonic crystal cavity. Opt. Express 20, 20884–20893 (2012). 23037212 10.1364/OE.20.020884

[b34] Wang J. *et al.* Reconfigurable radio-frequency arbitrary waveforms synthesized in a silicon photonic chip. Nat. Commun. 6, 5957 (2015). 25581847 10.1038/ncomms6957 PMC4354206

