# Supplementary material for: Ultra High-Speed Radio Frequency Switch Based on Photonics
Source: Sci Rep. 2015 Nov 26;5:17263. doi: 10.1038/srep17263 (PMC4660296; doi:10.1038/srep17263)
Supplement: Supplementary Information [file srep17263-s1.pdf]

Ultra High-Speed Radio Frequency Switch Based on Photonics

Jia Ge and Mable P. Fok*
Lightwave and Microwave Photonic Laboratory, College of Engineering, The University of Georgia
**Corresponding author: mfok@uga.edu*

Supplementary information

Specifications of major equipment and components:

DDMZM: FUJITSU FTM 7921ER 10-Gb/s Dual Driver Mach-Zehnder modulator.
PM: EOSPACE LN 52S 10-GHz phase modulator.
DFB: ILX Lightwave 79800, power fluctuation is less than 0.01 dB, wavelength fluctuation is less than 0.001 nm.
OSA: APEX AP2040A Optical Spectrum Analyzer, resolution 0.8 pm.
OSC: Agilent 86100D 30-GHz Dual Channel Electrical Oscilloscope.
SG: Agilent N5183A 20-GHz Signal Generator.
NA: Agilent E5071C 300 kHz ~ 20 GHz RF Network Analyzer.
RF Amplifier: JDSU H301-1210 10-Gb/s Modulator Driver and SHF 115 AP 20 GHz Broadband Amplifier.
RF 90° Coupler: RF-LAMBDA RFHB02G18GVT 18-GHz 90º Hybrid Coupler.
RF Spectrum Analyzer: Agilent N9020A 26.5 GHz Spectrum Analyzer.
